# Preliminary Stiffness-Driven Redesign of a Laminated Prosthetic Component Using Additive Manufacturing

**DOI:** 10.3390/polym15020346

**Published:** 2023-01-09

**Authors:** Luca Michele Martulli, Riccardo Sala, Gennaro Rollo, Milutin Kostovic, Marino Lavorgna, Andrea Sorrentino, Emanuele Gruppioni, Andrea Bernasconi

**Affiliations:** 1 Department of Mechanical Engineering, Politecnico di Milano, Via La Masa 1, 20156 Milano, Italy; 2 National Research Council (CNR), Polymer, Composites and Biomaterials Institute, Via Previati 1/E, 23900 Lecco, LC, Italy; 3 Istituto nazionale Assicurazione Infortuni sul Lavoro (INAIL), Centro Protesi Inail, Via Rabuina 14, 40054 Vigorso di Budrio, BO, Italy

**Keywords:** 3D printing, composite materials, fibre-reinforced polymers, prosthesis, application, numerical modelling

## Abstract

Three-dimensional printed polymers offer unprecedented advantages for prosthetic applications, namely in terms of affordability and customisation. This work thus investigates the possibility of designing an additively manufactured prosthetic foot using continuous fibre-reinforced polymers as an alternative to composite laminate ones. A numerical approach was thus proposed and validated as a possible design tool for additively manufactured composite feet. This approach was based on explicit separate simulations of the infill, aiming to capture its homogenised engineering constants. The approach was validated on simple sandwich specimens with a different infill geometry: stiffness predictions were within the experimental standard deviation for 3D simulations. Such an approach was thus applied to redesign a laminated component of a foot prosthesis inspired by a commercial one with new additive technology. The new component was about 83% thicker than the reference one, with 1.6 mm of glass fibre skins out of about 22 mm of the total thickness. Its stiffness was within 5% of the reference laminated one. Overall, this work showed how additive manufacturing could be used as a low-cost alternative to manufacturing affordable prosthetic feet.

## 1. Introduction

Prosthetic feet have been widely studied and used as replacements for lower limbs [1]. Historically, the most common type of prosthetic foot was the solid ankle cushioned heel (SACH) type. These early types of prostheses feature a rigid keel, which provides stability in the mid-stance phase of the gait cycle, but limited mobility and comfort. The more recent energy-storing-and-returning (ESAR) prostheses overcame these limitations by introducing a flexible keel; this allows the prostheses to store or release elastic energy during the gait cycle when needed [1,2,3]. Many of these prostheses are currently made of carbon fibre-reinforced polymers (CFRPs) via lamination. These materials and manufacturing techniques allow for lightweight, high-strength, and flexible structures [4,5]. However, the lamination process imposes severe limitations on the customisation of the prosthesis, and only standard sizes are available. Moreover, carbon fibre laminates are usually expensive materials. As a result, composite prosthetic feet are expensive devices. Considering that higher lower limb amputations are more frequent in lower-income areas, lowering the costs of these devices is of extreme importance [6,7].

The additive manufacturing (AM) of prostheses is one of the most promising manufacturing techniques to overcome these issues [8,9]. First of all, AM is considered a relatively cost-effective solution, thanks to reduced material waste and a mould-less working principle [10,11,12]. Moreover, it allows for an unprecedented freedom of design to be achieved for complex geometries with a relatively short lead time [10,11,12,13]. Combined with composite materials, AM can be effectively used for light structures with fair mechanical properties [10,11,12].

Several composite materials formulations can be used together with additive manufacturing [10,11,12]. One of the main distinctions that can be made between these formulations is based on the continuous or discontinuous nature of the reinforcement. Continuous fibres offer the highest contribution to the mechanical performance of 3D printed parts but impose additional design limitations [14]. The most commonly adopted fibres are carbon, Kevlar, and glass [10,11], in this order of mechanical properties and price (from highest to lowest). Short-fibre 3D-printed composites offer mechanical properties that are significantly lower but still higher than neat polymers [10,11,12,15]. Their cost is also generally in between the two.

The applications of AM for prosthetic feet are present in the literature [16]. Yap and Renda [17] designed and manufactured a low-cost 3D-printed SACH foot. The foot was printed on a hobby-level printer with polylactic acid (PLA). Failures during testing led to the implementation of metal rods. After this, the foot was able to sustain the weight of a person of 75 kg. Rochlitz et al. [18,19] used ABS to design and 3D-print an ESAR prosthetic foot. The design involved several FE analyses to ensure the foot could hold a maximum load of 1000 N. The authors showed the correct energy storage and release functionality for a 60 kg patient.

The possibility of printing complex geometries also allowed the exploitation of topology optimisation for weight reduction. Tao et al. [20] used a desktop 3D printer with PLA to design and manufacture a prosthetic foot. The authors designed the foot by topology optimisation techniques combined with finite element (FE) analysis. A similar approach was adopted by Vijayan et al. [21]. Both works showed excellent weight reduction capabilities. The high design flexibility allowed by AM also inspired some authors to tentatively reproduce the flexible joints of the foot. Auxetic sub-structures were considered for both the tow joint [22] and the heel [23]. Other applications include the integration of optical fibres as strain-sensing devices [24].

Overall, while the application of AM into prosthetic feet has shown promising results, significant work is still required for the product to reach the market. In particular, the works reported above adopt unreinforced polymers or short fibre-reinforced polymers. These materials cannot match the mechanical properties of laminated carbon fibre composites [10]; indeed, the aforementioned works deal mainly with children or patients with low mobility. As mentioned, continuous fibre-reinforced polymers have been recently introduced in the additive manufacturing sector. These materials allow for a significant improvement in both stiffness and strength [10]. Therefore, the aim of this work is to design an additively manufactured prosthetic foot component that matches the stiffness of a laminated CFRP one. To achieve this goal, AM technology was exploited by using continuous fibre-reinforced polymers in combination with 3D-printed composite sandwich structures [25]. To allow for the efficient and correct modelling of the infill core to be achieved, a numerical homogenisation technique was tentatively adopted and validated against experimental data. Then, a reference prosthesis component was selected and redesigned to exploit the AM capabilities. The objective was to match the reference component’s stiffness since this is the determining parameter affecting the comfort of use [26]. This work thus shows that 3D-printed prosthetic feet can possibly be a future low-cost alternative to laminated CFRP ones.

## 2. Materials and Methods

### 2.1. Specimens Manufacturing

The 3D printer adopted in this work was the Markforged Onyx Pro [27]. All specimens were printed using Onyx: a micro-carbon fibre-reinforced polyamide developed and supplied by Markforged [27]. The material parameters reported in the supplied datasheet are reported in Table 1. The data regarding continuous glass fibres are also reported and will be used for the numerical analysis.

**Figure 1:**
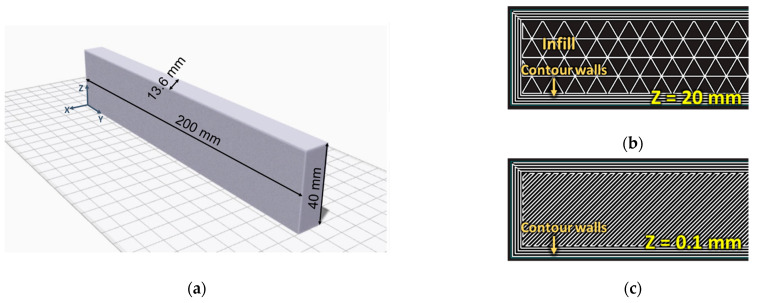
(**a**) 3PB specimen on a printing bed, (**b**) Central layers (with a triangular infill) and (**c**) Top/bottom layers.

Three-point bending (3PB) specimens were manufactured, as they more closely reproduce the foot prosthesis loading conditions (see Section 2.5). The composite foot prosthesis requires to be printed on a side to allow the placement of a contour of continuous glass fibres. This strategy was also adopted for the 3PB specimens to allow for a correct representation of the prosthesis component loading conditions. Figure 1a shows a 3PB specimen on the printing bed, as shown by the Markforged proprietary slicer software Eiger; the dimensions and coordinate system used in this work are also reported. The XY section is made of an infill and four concentric Onyx rings, as shown in Figure 1b. Two layers with a ±45° raster orientation were printed on the top and bottom of the specimens, perpendicularly to the Z axis with no infill to enclose the whole volume; these are shown in Figure 1c. The filament height and width were 0.2 mm and 0.4 mm, respectively.

The Markforged Onyx Pro allows for the printing of different infill shapes and densities. To allow the evaluation of different infill geometries and to have different validation cases for the considered approach, two infills were considered:

Triangular infill with 45% density. This will be referred to as “T45” in this work.

Rectangular infill with 20% density. This will be referred to as “R20” in this work.

Overall, the two specimen groups were manufactured and tested, differing only for the type of infill.

### 2.2. Three Point Bending Tests

Three-point bending tests were performed according to ASTM D7264-07 [28]. Five specimens per group were tested. The machine used was an MTS RF/100 equipped with a 100 kN load cell. A loading rate of 2 mm/min was chosen.

All specimens were tested using three different span lengths, namely 160 mm, 120 mm, and 80 mm. This was conducted to further expand the range of validation cases available for the considered modelling approach. The specimen’s stiffness, calculated as the slope of the load–displacement curve, was calculated in the skins’ strain range between 0.1% and 0.3%, as suggested by the standard [28]. To this end, the maximum mid-span deflection *δ_max_* was calculated via Equation (1) [28]:

(1) $$\delta_{max}=\frac{\varepsilon_{max}\cdot L^{2}}{6h}$$

where *ε_max_* is the maximum strain, set to 0.3%, *L* is the span length, and *h* is the thickness of the specimens. The resulting theoretical maximum vertical displacements and the applied ones are reported in Table 2. Table 2 also reports the calculated maximum strains at the actual applied vertical displacements. As shown, the maximum strain in the outer layers is significantly smaller than the 1.7% yield strain reported in the supplier datasheets [27]. Therefore, no damage or plasticity is expected to be introduced in the specimens during testing, which justifies the use of the same specimens for different span lengths.

### 2.3. Core Homogenisation through Explicit Modelling

The explicit simulation of the infill geometry is an inefficient approach to FE simulations [25]. This is due to both an increased computational time for each simulation performed and an increased modelling effort in the pre-processing phase. For this reason, a numerical investigation was performed to obtain the equivalent homogenised, in-plane elastic constants of the infill. This numerical investigation was based on several explicit simulations of the infill geometry alone.

**Figure 2:**
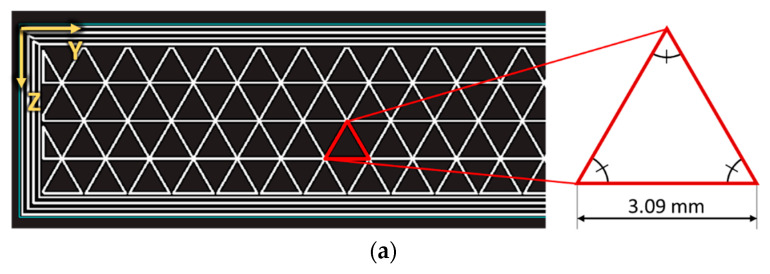

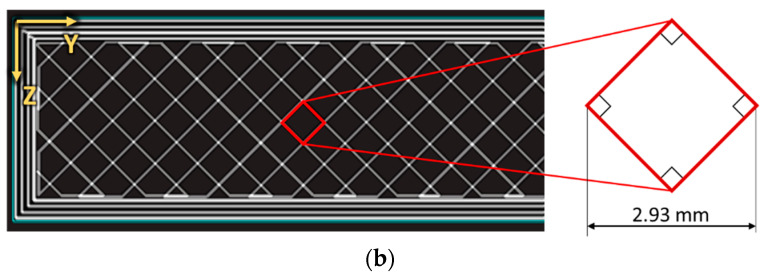
Extracted geometries of the unit-cells for the (**a**) T45 infill and the (**b**) R20 infill. Note that the for the R20 infill, the printer prints the infill with unidirectional layers (+45° or −45°); therefore, the figure (**b**) was obtained as the superposition of two subsequent layers.

To correctly model the infill geometries, the dimensions of the unit cells comprising the considered infills were extracted from the slicing software Eiger. The results of this operation are shown Figure 2a,b and showcase the dimensions of the unit cells composing the T45 and R20 infill, respectively.

**Figure 3:**
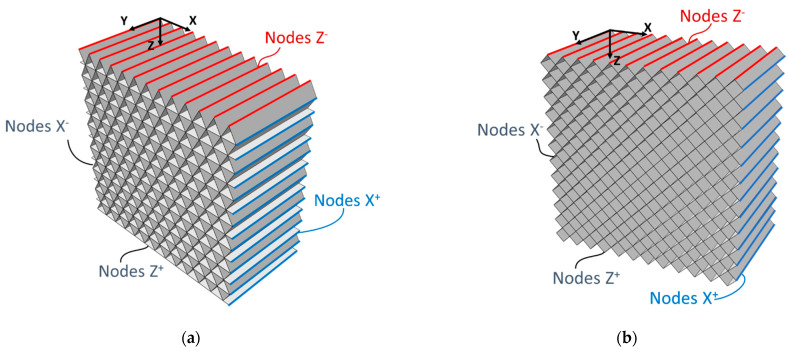
(**a**) T45 and (**b**) R20 explicit models.

Based on the extracted geometric information, 3D shell models were created on Abaqus [29] to represent the infills. The considered models included many unit cells in order to reduce any boundary effect. Figure 3 showcases both the T45 and R20 geometries modelled in Abaqus. The in-plane dimensions are provided by the dimensions of the unit cells; an arrangement of 12 × 14 unit cells and of 10 × 10 unit cells was modelled for the T45 and the R20, respectively. The out-of-plane dimension was set to 20 mm. The shell thickness was set to 0.4 mm: the same as the width of the filaments. The local orientation was assigned, with direction one along the edge of the infills. The adopted material parameters are reported in Table 3.

Three simulations were performed for the T45 geometry to search for E_1_, E_2,_ and G_12_. For the R20 geometry, two simulations were performed to compute E_1_ and G_12_ because the geometry was symmetric by rotation: E1 and E2 simulations would thus be equivalent. Figure 3 highlights the group of nodes at the boundaries of the geometries. These are used to apply the boundary conditions in the different simulations for the evaluation of E_1_, E_2,_ and G_12_. The boundary conditions applied to each case are reported in Table 4. Moreover, all nodes lying on the plane Y = 0 (see Figure 3) were prevented from moving along the Y axis. Quadratic shell elements S8R, with a global mesh size of 1 mm, were used for all simulations because shell elements proved to be effective for modelling this type of core geometry [25].

### 2.4. Three-Point Bending Simulations

**Figure 4:**
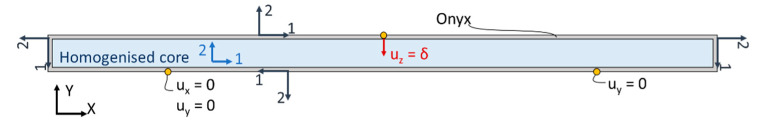
Two-dimensional 3PB simulations with a 160 mm span length. Axes 1–2 indicate local orientation.

Two different approaches were adopted for the simulations of the 3PB specimens. First of all, 2D simulations were performed with a homogenised core. Figure 4 shows the modelled 2D specimens with a span length of 160 mm. As shown, a contour layer of Onyx simulates the outer skins of the specimen; a local orientation is assigned to the layer and is defined via the tangential and normal directions. The elastic properties of the core are the ones obtained through the core homogenisation step previously described (see Section 3). The local orientation is aligned with the global reference system (see Figure 4). Moreover, two reference points were used to apply the boundary conditions, simulating the support rollers; a third reference point was used to apply an imposed displacement. The applied displacements are the same as those applied during the tests, as reported in Table 2. Note that the different span lengths are simulated by moving the two lower reference points closer together. Finally, the adopted material constants are reported in Table 3.

**Figure 5:**
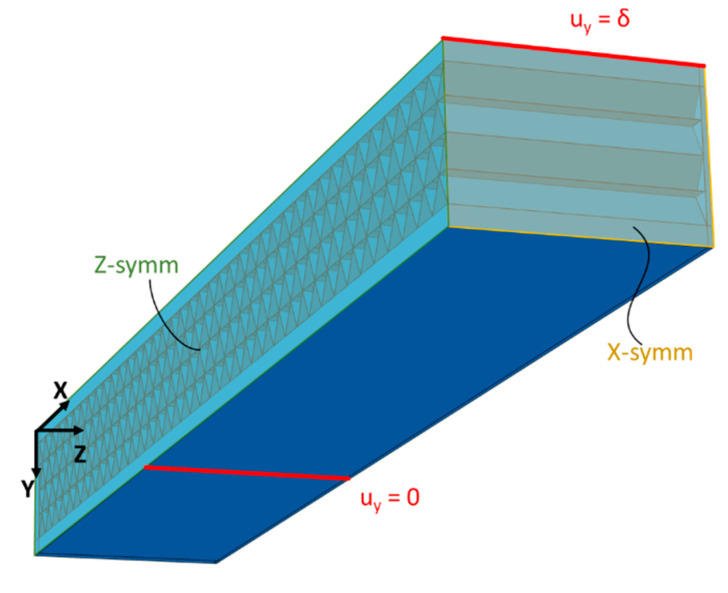
Three-dimensional 3PB simulation for the T45 core with a 160 mm span length. δ refers to the applied displacement reported in Table 2 for the different simulations.

Three-dimensional simulations of the 3PB specimens, with an explicit core representation, were also performed. This was performed to compare the homogenised core approach with an additional modelling strategy which is closer to reality but less applicable to full structures. Only a quarter of the 3PB specimens were modelled to reduce the computational time required. The exemplificative case of the T45 core, with a 160 mm span, is shown in Figure 5. Boundary conditions are also reported. The material properties are the ones reported in Table 3.

**Figure 6:**
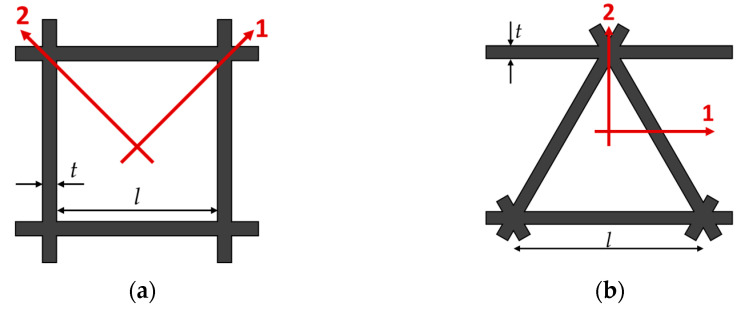
Core geometries for the analytical evaluations: (**a**) Rectangular and (**b**) Triangular.

Finally, the cores’ engineering constant was also evaluated analytically, using Equations (2) and (3) for a rectangular and a triangular core, respectively [30]. In particular, referring to Figure 6, *E*_1_ and *E*_2_ are Young’s moduli in directions one and two, respectively; *E_onyx_* is Young’s modulus of the Onyx, and *t* and *l* are defined in Figure 5.

(2) $$\frac{E_{1}}{E_{onyx}}=\frac{E_{2}}{E_{onyx}}=2{\left(\frac{t}{l}\right)}^{3}$$

(3) $$\frac{E_{1}}{E_{onyx}}=\frac{E_{2}}{E_{onyx}}=1.15\frac{t}{l}$$

### 2.5. Reference Prosthesis Simulation

**Figure 7:**
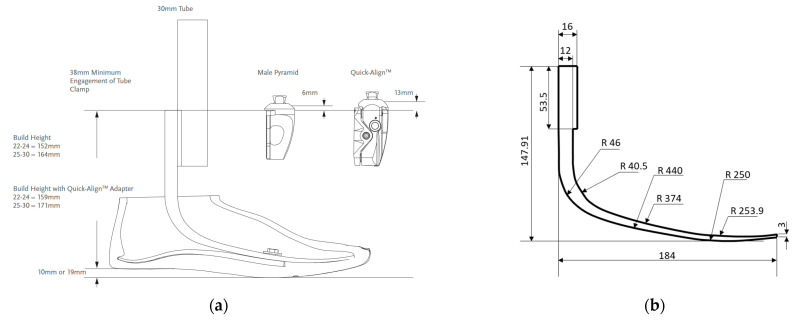
(**a**) The Össur VARI–FLEX^TM^ design taken from [32] and (**b**) the inspired laminated component.

The objective of this work was to design an additively manufactured glass-reinforced prosthetic component with a stiffness comparable to that of a commercial one. Glass fibres were preferred to carbon fibres to keep the cost of the new prosthesis as low as possible. Prosthetic feet, currently, are often manufactured via the lamination of continuous fibre-reinforced composites [31]. To set a benchmark stiffness, a component of a commercially available laminated carbon fibre prosthesis was first simulated. The reference prosthesis was inspired by the Össur VARI-FLEX^TM^, represented in Figure 7a [32]. As shown, the prosthesis is mainly composed of two laminated components connected by bolts. Since no information was available on the bolted connection, it was decided to model only the spring. The extracted simulated component is shown in Figure 7b.

**Figure 8:**
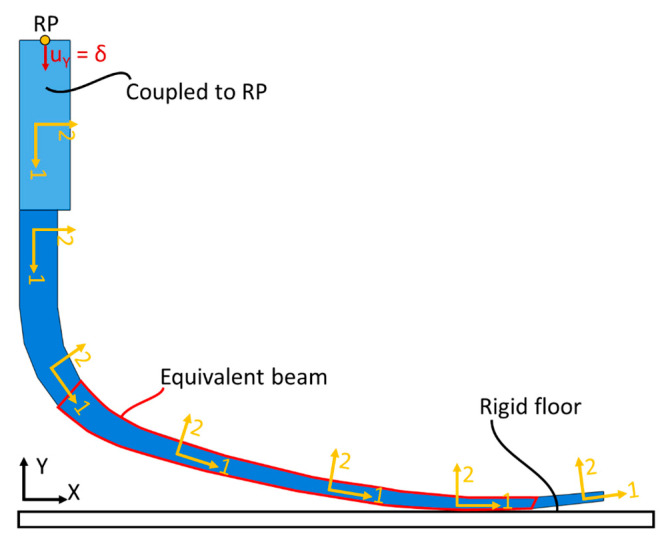
Model of the reference component.

A 2D FE simulation was performed on the reference component. It was modelled as a quasi-isotropic carbon fibre laminate whose properties are reported in Table 5. The table reports the elastic properties in the local coordinate system; the local orientation of the material along the prosthesis is shown in Figure 8. Moreover, Figure 8 also shows the boundary conditions applied to the reference prosthesis. In particular, the upper thicker portion of the prosthesis is coupled to a reference point with a kinematic coupling; the reference point is then lowered by 10 mm. A rigid analytical rectangular surface is then created to simulate a rigid floor. A frictionless contact is then created between the lower surface of the prosthesis and the floor.

## 3. Results and Discussions

### 3.1. Homogenisation Results

The engineering constants obtained from the explicit simulations are reported in Table 6. As shown, the E_1_ simulations led to the estimation of Poisson’s ratios of the cores as well. Moreover, a slight anisotropy was observed for the T45 core. While the triangular honeycombs are known to have isotropic in-plane properties [30], the difference between the computed E_1_ and E_2_ is smaller than 5%, and thus, it was considered acceptable. The analytical results are also reported in Table 7. As shown, the results demonstrate quite a good agreement between the two approaches. The 30% discrepancy obtained for the R20 core is more likely due to the very low value of its modulus, which makes a small difference of 9 MPa significant.

**Figure 9:**
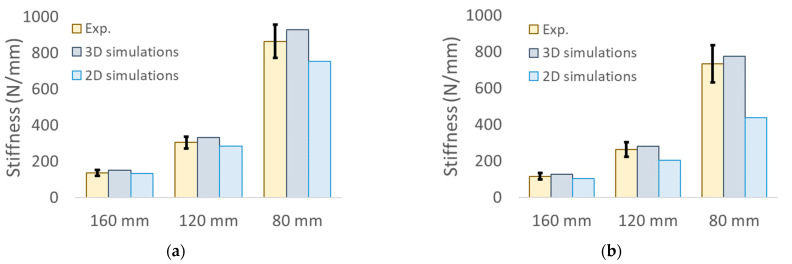
Stiffness results for the (**a**) T45 and the (**b**) R20 specimens.

Figure 9a,b showcases the comparison between the experimentally measured and the computationally calculated stiffnesses for the T45 and the R20 specimens, respectively. The numerical values are also reported in Table 7. As shown, the 2D simulations consistently show some degree of underpredictions, especially for the low span length. This was expected because the 2D analyses did not simulate the presence of the lateral walls, which have a non-negligible impact on the specimens’ stiffness. This is also supported by the fact that the full 3D simulations show a very good agreement with the experimental results.

Considering the results reported above and the fact that the considered prosthetic component presents a relatively long span (see Section 2.5), it was decided that the obtained engineering constants for the design of the component should be used.

### 3.2. Design and Optimisation of the 3D Printed Components

Laminated carbon fibre composites generally display better mechanical properties than 3D-printed glass fibre ones. Therefore, the newly designed 3D-printed component must be significantly thicker than the reference one. A thicker 3D-printed composite would also behave as a composite sandwich component, which is generally a very efficient lightweight structure [25].

A preliminary analytical optimisation was thus performed to identify the best geometrical parameters for the new component. The complex shape of the laminated component prevents an analytical optimisation over its whole shape. For this reason, the analytical optimisation was performed on an equivalent cantilever beam which was representative of a portion of the component highlighted in Figure 9. The highlighted portion indeed behaves as a cantilever beam, whereas the stiffer vertical portion behaves similar to a constraint, and the contact with the floor introduces the load.

For a cantilever sandwich beam, the skins and the core determine the bending and shear rigidities, respectively. Therefore, the bending and shear rigidities *K_b_* and *K_s_* can be obtained via Equations (4) and (5), respectively:

(4) $$K_{b}=\frac{E_{f}\cdot b\cdot t\cdot c^{2}}{2}$$

(5) $$K_{s}=\frac{G_{c}\cdot b\cdot {\left(c+t\right)}^{2}}{c}$$

where *E_f_* and *G_c_* are the elastic moduli of the fibres and the shear modulus of the core, respectively; *b*, *t,* and *c* are the width of the panel, the thickness of the skins, and the thickness of the core, respectively. Moreover, the beam flexibility, namely the ratio of the tip displacement *δ* at a given load *P* over that load, and weight *W* are calculated as:

(6) $$\frac{\delta }{P}=\frac{l^{3}}{3K_{b}}+\frac{l}{K_{s}}$$

(7) $$W=2\rho_{s}bltg+\rho_{c}blcg$$

where *l* is the length of the beam, *g* is the acceleration of gravity, and *ρ_s_* and *ρ_c_* are the densities of the skins and core, respectively. Note that the cores’ densities were calculated using the volume occupied by the Onyx in a single cell times the Onyx density over the total volume of the cell. Moreover, the shear moduli of the cores were obtained via the homogenisation procedure described in Section 3. Regarding the skins, the properties were assumed to be equal to those of the glass fibres (see Table 1).

The design variables of the optimisation procedure are *t/l* and *c/l*, namely the thicknesses of the skins and the core when normalised with the beam of the length. The optimisation constraint imposes that the new design has the same stiffness as the reference prosthesis, which is obtained via the FE simulations described in Section 2.5. The objective of the optimisation was to minimise the weight. The parameters adopted are reported in Table 8. Two optimisations were performed, considering a T45 and an R20 core. Therefore, two different designs of the same component will be obtained and further evaluated.

**Figure 10:**
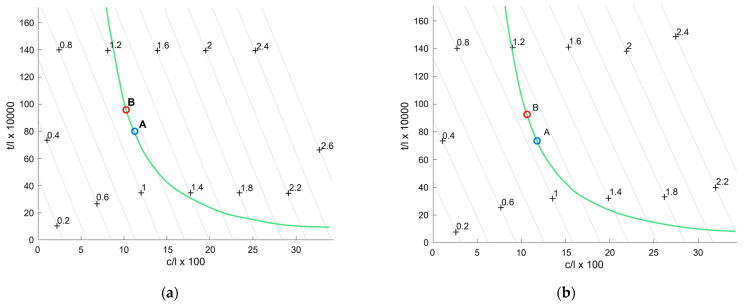
Optimisation results considering a (**a**) T45 and a (**b**) R20 core.

The results of the optimisations are reported in Figure 10a,b for the T45 and R20 cores, respectively. In the graphs, the iso–weight curves appear linear, while curves relative to the stiffness requirements are hyperboles. In both figures, the points labelled A show the optimal design variables to minimise the weight. The relative results are reported in Table 9. As shown, the skin’s thickness is not a multiple of 0.8 mm, which is the width of the glass fibre filaments specified by the supplier [27]. Both points A are, therefore, unfeasible solutions. A more realistic design had to be selected on the same stiffness curve. Points B were thus considered, the dimensions of which are also reported in Table 9; the weight increase with respect to the optimal points was found to be less than 2% in both cases.

**Figure 11:**
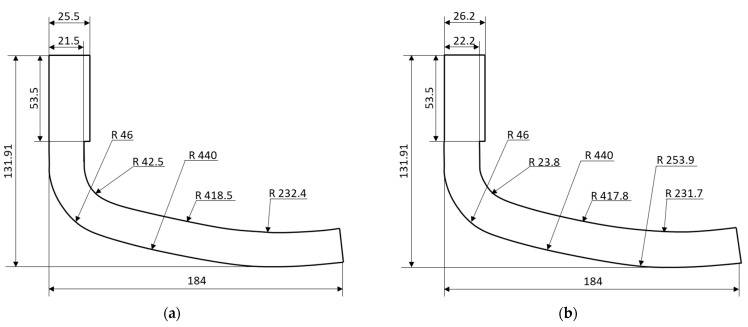
Optimised geometry of the prosthesis component considering a (**a**) T45 and a (**b**) R20 core.

The resulting designs of the 3D-printed prosthetic component, considering a T45 or an R20 core, are reported in Figure 11a,b, respectively. Note that these were obtained by leaving the shape of the lower surface unaltered, using the updated core and skin thicknesses.

Two-dimensional FE simulations were then performed on the newly designed prostheses to compare them with the reference laminated one. The boundary conditions, mesh size, and types are the same as those used for the reference prosthesis simulations described in Section 2.5. The material parameters adopted for the core are the ones obtained from the homogenisation procedure and reported in Table 6; the glass fibre properties are reported in Table 10.

**Figure 12:**
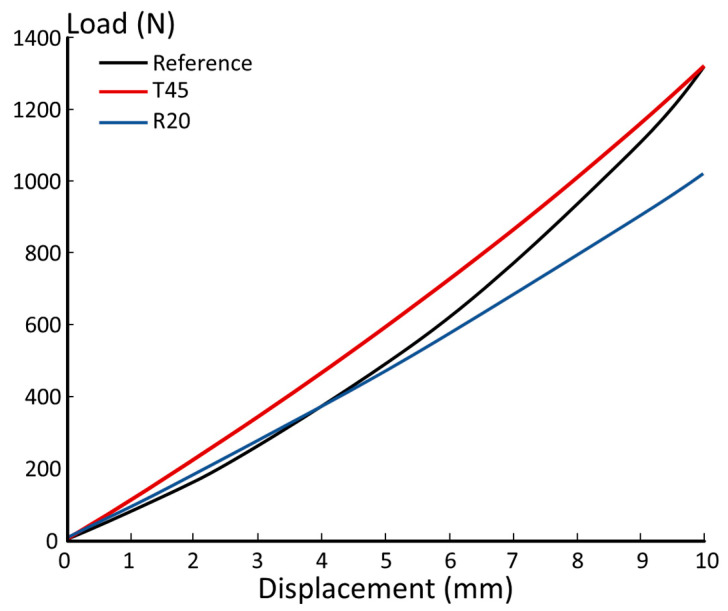
Load–displacement curves from the FE simulations.

The load–displacement curves obtained from this comparison are reported in Figure 12. As shown, both 3D-printed prosthesis components show a stiffness that is comparable to that of the laminated reference.

### 3.3. 3D Printed Prosthesis Components and Further Design Work

The FE analyses performed in this work show that 3D-printed components can meet the stiffness requirements of prosthetic feet. This is an encouraging result because stiffness is one of the major requirements for this kind of structure [26]. Moreover, further improvements can still be applied to the proposed designs. First of all, glass fibres were considered in this work as a continuous reinforcement instead of carbon fibres to reduce the cost of the prosthetic device. However, the use of the more performing carbon fibre can further increase the mechanical properties of a prosthetic component, thus allowing smaller thicknesses and a slenderer shape.

Another possible improvement to a 3D-printed prosthesis would be the manufacturing of an integrated prosthesis. As shown in Figure 8, the reference prosthesis is composed of two main components: a common solution for laminated prostheses [32]. However, the increased manufacturing flexibility allowed by AM can be exploited to design a one-component prosthesis. Such prosthesis would thus integrate the functionalities of adequate contact to the ground and the connection to the upper pylon. This would further reduce the cost of the final products, thanks to reduced assembly costs.

Finally, while the potential of an additively manufactured foot is demonstrated here, further work is required for it to reach the market. In particular, it must be proven that the prosthetic component can also resist fatigue loads in both standard and extreme environmental conditions (considering a wide range of temperatures and humidity). This requires further work on the different 3D-printed materials because their fatigue characterisation and modelling are still an active research field [14,33,34]. To reach commercialization, further analysis is needed in the framework of the biomechanical behaviour of the prosthesis, similar to the analysis of the roll-over shape and the assessment of the energy storage and releasing capability.

## 4. Conclusions

The present work investigated the possibility of designing an additively manufactured prosthetic foot with the same stiffness as that of a composite laminated one. It was decided to consider a 3D-printed sandwich structure because these structures generally show good bending responses.

To correctly model the infill elastic behaviour, a numerical homogenisation procedure was adopted. The homogenised engineering constants were extracted via the explicit modelling of the actual infill geometry. To validate this procedure, experimental tests were conducted on three points for the bending specimens.

Once a satisfying modelling procedure was identified, it was adopted to design a prosthetic foot component. First of all, a reference component inspired by a commercially available one was considered as a benchmark, and it was simulated to compute its stiffness. The reference component was made of laminated carbon fibre composite. Then, new designs of an additively manufactured component with the same stiffness as the reference were obtained via an optimisation procedure. This optimisation aimed to minimise the prosthesis weight. Once the new designs were obtained, their mechanical response to a vertical load (simulating the weight of a person) was computed via FE analysis, showing a good agreement with the reference structure. Therefore, this work shows promising results in the adoption of continuous fibre-reinforced additively manufactured polymers for low-cost prosthetic feet.

## Figures and Tables

**Table 1:** Properties of the Onyx and of continuous glass fibre supplied by Markforged [27].

Property	Onyx	Glass Fibre
Tensile Modulus (GPa)	2.4	21
Tensile stress at yield (MPa)	40	-
Tensile stress at break (MPa)	37	590
Tensile strain at break (%)	25	1.1
Density (g/cm^3^)	1.2	1.5

**Table 2:** Theoretical (calculated via Equation (1)) and applied maximum vertical displacement during the three point bending tests.

Parameter	L = 160 mm	L = 120 mm	L = 80 mm
Theoretical max. vertical displacement (Equation (1))	0.94 mm	0.52 mm	0.24 mm
Applied max. vertical displacement	1 mm	0.65 mm	0.3 mm
Maximum strain at applied vertical displacement	0.32%	0.375%	0.375%

**Table 3:** Onyx engineering constants used in the simulations.

Parameter	Value
E_1_ (GPa)	2.4
E_2_ (GPa) ^1^	1.7
E_3_ (GPa) ^1^	1.7
ν_12_ ^2^	0.29
ν_13_ ^2^	0.29
ν_23_	0.35
G_12_ (MPa) ^2^	190
G_13_ (MPa) ^2^	190
G_23_ (MPa) ^3^	658

^1^ Transverse moduli were considered equal to that of unreinforced Nylon, as reported in [27]. ^2^ From [25]. ^3^ Considering G_23_ = E_2_/(2 + 2∙ν_23_).

**Table 4:** Applied boundary conditions to the different simulations (u_x_, u_y,_ and u_z_ refer to displacements along *X*-axis, *Y*-axis, and *Z*-axis, respectively).

	T45	R20
E1	Nodes X^+^: u_x_ = 1 mm	Nodes X^+^: u_x_ =1 mm
	Nodes X^−^: u_x_ = 0 mm	Nodes X^−^: u_x_ =0 mm
	Nodes Z^−^: u_z_ = 0 mm	Nodes Z^−^: u_z_ = 0 mm
E2	Nodes Z^+^: u_z_ = 1 mm	-
	Nodes Z^−^: u_z_ = 0 mm	
	Nodes X^−^: u_x_ = 0 mm	
G12	Nodes X^+^: u_x_ = 0 mm	Nodes X^+^: u_x_ = 0 mm
	Nodes X^+^: u_z_ = 1 mm	Nodes X^+^: u_z_ = 1 mm
	Nodes X^−^: u_x_ = 0 mm	Nodes X^−^: u_x_ = 0 mm
	Nodes X^−^: u_y_ = 0 mm	Nodes X^−^: u_y_ = 0 mm

**Table 5:** Properties of the quasi-isotropic carbon fibre laminate used in the reference component simulation. All moduli are in MPa and Poisson’s ratios are dimensionless. The coordinate system considered is the local one.

E_1_	E_2_	E_3_	ν_12_	ν_13_	ν_23_	G_12_	G_13_	G_23_
60,000	10,500	60,000	0.3	0.3	0.175	5000	5000	3500

**Table 6:** Results of the explicit homogenisation procedure.

	T45		R20	
	FEM	Analytical	FEM	Analytical
E_1_ (MPa)	400	410	23	32
E_2_ (MPa)	419	410	-	32
ν_12_	0.318	-	0.476	-
G_12_ (MPa)	109	-	45	-

**Table 7:** Experimentally measured and computed stiffnesses (all in N/mm). “±” denotes standard deviation.

	T45			R20		
Span	Exp.	3D FE	2D FE	Exp.	3D FE	2D FE
80 mm	864 ± 91	927	752	734 ± 102	774	437
120 mm	304 ± 33	332	284	263 ± 41	281	202
160 mm	136 ± 16	150	131	116 ± 19	126	103

**Table 8:** Parameters used in the optimisation procedure.

Parameter	Description	Value	
*l*	Beam length	175 mm	
*b*	Beam width	50 mm	
*P/δ*	Reference prosthesis stiffness	131.6 N/mm	
*E_s_*	Skins’ elastic modulus	21 GPa	
*G_c_*	Cores’ shear modulus	109 MPa (T45)	45 MPa (R20)
*ρ_s_*	Density of the skins	1500 kg/m^3^	
*ρ_c_*	Density of the cores	477 kg/m^3^ (T45)	418 kg/m^3^ (R20)

**Table 9:** Numerical results of the optimisation procedures.

	Optimal Point A		Realistic Point B	
	Skins Thickness *t*	Core Thickness *c*	Skins Thickness *t*	Core Thickness *c*
T45	1.4 mm	19.5 mm	1.6 mm	18.3 mm
R20	1.3 mm	20.6 mm	1.6 mm	19.0 mm

**Table 10:** Properties of glass fibre material used in the prosthesis simulations. All moduli are in MPa and Poisson’s ratios are dimensionless. The coordinate system considered is the local one.

E_1_	E_2_	E_3_	ν_12_	ν_13_	ν_23_	G_12_	G_13_	G_23_
21,000	2318	2318	0.31	0.31	0.43	859	859	819

## Data Availability

Data will be available on request.
